# Therapeutic Liposomal Vaccines for Dendritic Cell Activation or Tolerance

**DOI:** 10.3389/fimmu.2021.674048

**Published:** 2021-05-13

**Authors:** Noémi Anna Nagy, Aram M. de Haas, Teunis B. H. Geijtenbeek, Ronald van Ree, Sander W. Tas, Yvette van Kooyk, Esther C. de Jong

**Affiliations:** ^1^ Department of Experimental Immunology, Amsterdam University Medical Center, Amsterdam Institute for Infection and Immunity, University of Amsterdam, Amsterdam, Netherlands; ^2^ Department of Molecular Cell Biology and Immunology, Amsterdam University Medical Center, Cancer Center Amsterdam, Amsterdam Institute for Infection and Immunity, Vrije Universiteit Amsterdam, Amsterdam, Netherlands; ^3^ Department of Otorhinolaryngology, Amsterdam University Medical Center, University of Amsterdam, Amsterdam, Netherlands; ^4^ Department of Rheumatology and Clinical Immunology, Amsterdam University Medical Center, Amsterdam Rheumatology and Immunology Center, University of Amsterdam, Amsterdam, Netherlands

**Keywords:** dendritic cell (DC), nanoparticle, liposomes, tolerance, activation, antigen, targeting, adjuvant

## Abstract

Dendritic cells (DCs) are paramount in initiating and guiding immunity towards a state of activation or tolerance. This bidirectional capacity of DCs sets them at the center stage for treatment of cancer and autoimmune or allergic conditions. Accordingly, many clinical studies use *ex vivo* DC vaccination as a strategy to boost anti-tumor immunity or to suppress immunity by including vitamin D3, NF-κB inhibitors or retinoic acid to create tolerogenic DCs. As harvesting DCs from patients and differentiating these cells *in vitro* is a costly and cumbersome process, *in vivo* targeting of DCs has huge potential as nanoparticulate platforms equipped with activating or tolerogenic adjuvants can modulate DCs in their natural environment. There is a rapid expansion of the choices of nanoparticles and activation- or tolerance-promoting adjuvants for a therapeutic vaccine platform. In this review we highlight the most recent nanomedical approaches aimed at inducing immune activation or tolerance *via* targeting DCs, together with novel fundamental insights into the mechanisms inherent to fostering anti-tumor or tolerogenic immunity.

## Introduction

The incidence and prevalence of cancer as well as several auto-immune, inflammatory and allergic conditions is on the rise (1, 2). While multiple treatment strategies exist for these conditions, the majority of them have side effects or other drawbacks. Chemotherapy is toxic to all dividing cells in the body, causing strong systemic side effects. Allergies are mostly treated by symptomatic drugs such as antihistamines and local and systemic corticosteroids. For some allergies, a disease-modifying treatment, allergen immunotherapy (AIT), is available but is not used very broadly (3). Although AIT is quite effective, it requires monthly injections or daily sublingual administration of allergen extract for at least 3-5 years. Moreover, it carries the risk for anaphylactic reactions. For autoimmune diseases so-called Disease Modifying Anti-Rheumatic Drugs (DMARDS) are often prescribed (4, 5). These therapies suppress a wider set of immune cells than the pathogenic players, increasing the risk for infections. Furthermore, treatment has to be continued throughout life, yielding no perspective of a cure (6). There has been tremendous progress in our understanding and harnessing of the immune system to treat these diseases. Immunotherapy is already used in the clinic to treat cancer and inflammatory diseases, but the reprogramming of the immune system to attack and eliminate the tumor or suppress inflammatory responses is also very attractive.

Dendritic cells (DCs) are key in initiating a proper anti-tumor response, as well as dampening adaptive immunity when tolerance to innocuous antigens or auto-antigens is needed (7, 8). DCs initiate an anti-tumor cascade by the uptake of particles derived from tumor cells and cross-presenting the tumor antigens on MHC-I for efficient activation of CD8+ T cell responses (9). Initiation of T_H_1 type CD4+ T cell responses *via* DC-derived cytokines such as IL-12 is a crucial component in the anti-tumor response, reinforcing the expansion of CD8+ T cells and licensing CTLs for (tumor) killing (10).

To foster central tolerance in cooperation with thymic epithelial cells, DCs contribute to the deletion of effector T cells in the thymus (11). Lack of co-stimulation by DCs in the periphery leads to anergy or apoptosis of effector T cells. A long reigning dogma proposed that DCs rather passively mediate tolerance *via* an immature or semi-mature state. Opposing this dogma, recent insights challenge the notion that immature DCs effectively promote steady-state tolerance *in vivo*, suggesting that both immunogenic and tolerogenic migratory DCs are ‘mature’ or activated, and clearly distinguishable by differential expression of quantitative and qualitative markers (12). Supporting this statement, DCs are known to actively induce tolerance *via* co-inhibitory signaling and tolerogenic cytokine production. Active engagement of co-inhibitory signals, such as programmed cell death-1 (PD-1), cytotoxic T-lymphocyte-associated antigen 4 (CTLA-4), and others by their respective receptors on DCs leads not only to anergy and effector T cell deletion, but also to the development of regulatory T cells (Tregs) and reverse signaling in DCs that reinforces their tolerogenic capacity (13). Similarly, distinct surface molecules, immunoregulatory enzymes and cytokines, such as indoleamine-2,3-dioxygenase (IDO), IL-10 and TGF-β produced by DCs can dampen effector T cells, and potently induce several subtypes of Tregs (14). With these strategies at hand tolerogenic DCs can contribute both to deletion of autoreactive T cells in autoimmunity and deletion of T_H_2 cells supporting allergic inflammation (15, 16). Moreover, DCs facilitate the development of regulatory B cells which produce more IL-10 for ameliorating autoimmune conditions as well as IgG4, essential for dampening pro-allergic responses (16–18). The versatile skills of DCs coordinating both tolerogenic and inflammatory immune responses make them an excellent target for novel therapies against cancer, autoimmune disease and allergies.

Unsurprisingly, DC-based therapies are now in clinical trial phases for the treatment of various forms of cancer and autoimmune disease (8, 15). A popular DC-based approach is *ex vivo* DC vaccination, a therapy in which patient monocytes or CD34+ progenitors are cultured together with DC activating adjuvants, or DC dampening anti-inflammatory adjuvants, and disease relevant antigen, for subsequent reinfusion in the patient (8, 15). Unfortunately, even though these therapies appear to be safe and well-tolerated, much needs to be done to increase therapeutic efficacy. One putative explanation for this observation is that *ex vivo* cultured DCs are largely monocyte-derived that differ from the naturally occurring DCs *in vivo (* 19). In addition, *ex vivo* DC therapy is costly and cumbersome, as cells have to be processed in a controlled, sterile lab environment.

**Figure 1:**
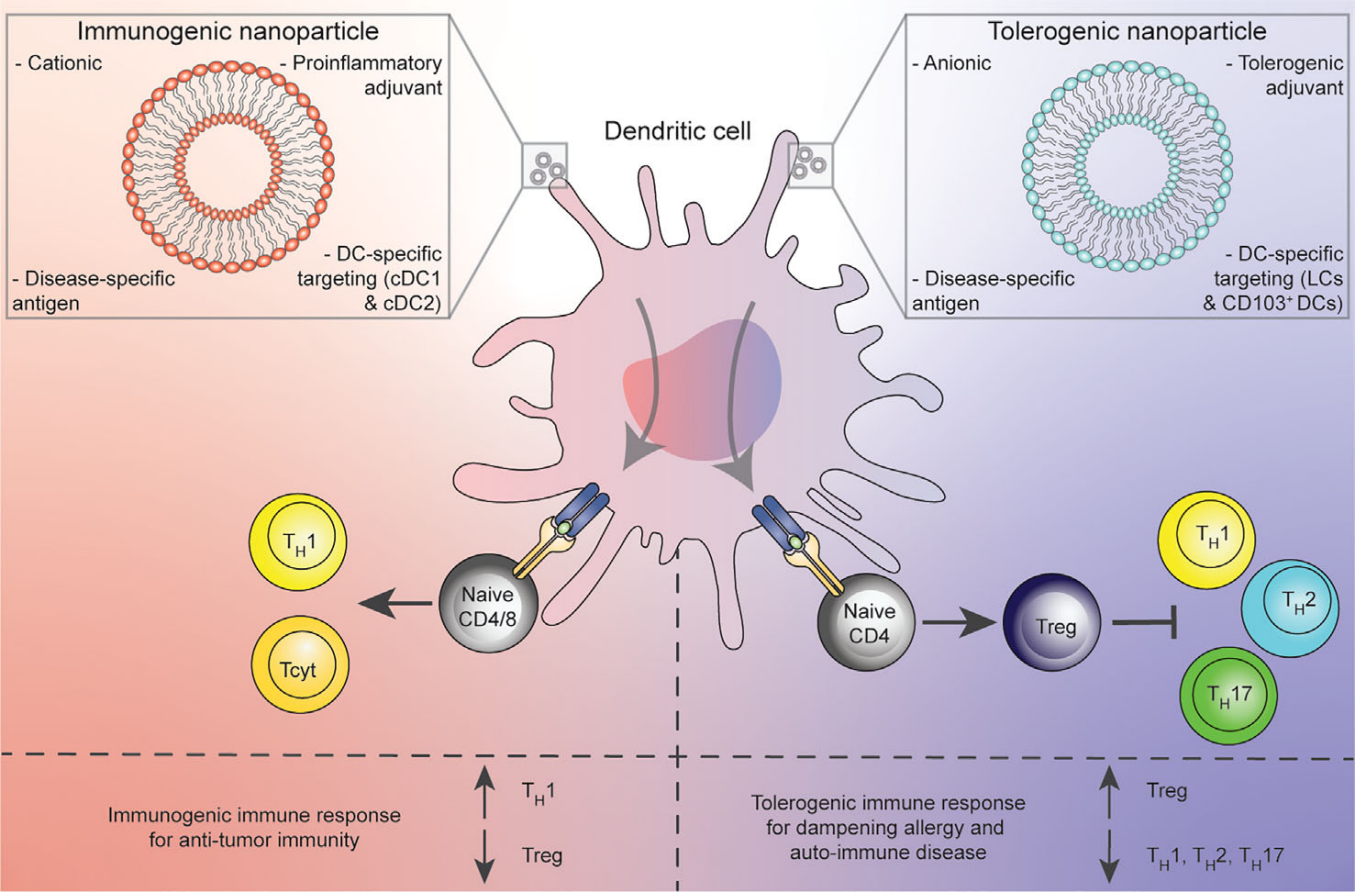
Concept of *in vivo* treatment of DCs with immunogenic (red) or tolerogenic (blue) nanoparticle platforms resulting in pro-inflammatory DCs that prime for T_H_1 or Tcyt polarization against cancer (left) or Tregs for the dampening of allergic and auto-immune conditions (right).

A radically different, promising approach is the targeting of DCs *in vivo*, *via* a therapeutic vaccination-like strategy (**Figure 1**). This approach has the advantage of bypassing costly *ex vivo* isolation and preparation of DCs and potentially provides opportunities of tissue-site targeting of multiple DC subsets in their natural environment (8). Furthermore, *in vivo* targeting platforms can be made available to a broad range of patients, as they are not donor dependent.

One type of treatment focusses on the targeting of DCs *via* antibody-antigen or glycan-antigen conjugates for routing to various surface receptors predominantly expressed on these antigen presenting cells (APCs), as recently reviewed in the context of cancer or immune tolerance elsewhere (20, 21).

A second strategy employs nanoparticles as vehicles for loading disease relevant antigen, adjuvant and targeting molecules to reach DCs *in vivo* (**Figure 1**). Nanoparticle platforms provide the pharmacological advantages of sequestering potentially toxic contents from undesired targets, and release of contents in a controlled fashion to increase bioavailability of compounds (22, 23). Besides these general advantages, a large body of literature states that various nanoparticles have *bona fide* adjuvant effects as they preferentially are engulfed by APCs (22). Although a wide variety of different nanoparticles for various purposes have been developed, we mainly focus on liposomes, nanoparticles composed of a lipid bilayer. Liposomes are not only already FDA approved, but are also highly flexible in that several of their characteristics, such as lipid composition, size, shape, electrical charge and rigidity can be modified (24). Furthermore, due to their chemical structure consisting of a lipophilic bi-layer and a hydrophilic core, liposomes also provide an ideal platform for uniting all desired components of an immune modulatory vaccine (disease relevant antigens, DC-targeting moieties and adjuvants) in one spatial compartment.

## DC Subsets for Anti-Cancer and Tolerogenic Immunomodulation

For *in vivo* targeting of DCs one needs to consider that *in-situ* DCs are comprised of a heterogenous mix of subpopulations, with indications of functional differences between the subsets. In humans, current nomenclature describes three major subtypes of DCs based on surface markers: conventional type 1 DCs (cDC1s or CD141+ DCs), conventional type 2 DCs (cDC2s or CD1c+DCs) and plasmacytoid DCs (pDCs) (25). One feature supporting an intrinsic inclination of these subsets to respond in a pro-or anti-inflammatory fashion to different pathogens is their differential expression of various pattern recognition receptors (PRRs) (26). cDC1s highly express toll-like receptors (TLRs) 3, 9 and 10 enabling recognition of intracellular dsRNA or DNA leading to production of type-I interferons and IL-12 (27). cDC2s express the full range of TLR1-9 and a wide range of C-type lectins (CLRs) equipping them with a broad toolkit to respond to various pathogens. pDCs highly express TLRs 7 and 9, leading to a swift type I and III interferon response *via* IRF7 and an efficient anti-viral reaction. cDC1s are classically described to be more apt at cross-presenting antigen to CD8+ T cells, yet can also potently silence these cells for tolerance, and cDC2s seem to effectively advance CD4+ T cell proliferation (26). Also, under inflammatory conditions monocytes can differentiate into monocyte-derived DCs (moDCs) (28). Recent research further subdivides and expands on the current populations of DCs, for example the cDC2-A (DC2), cDC2-B (DC3), and Axl+DCs (27, 29). It is however beyond the scope of this review to go into detail about all the classes and subdivision within the DCs, and we will therefore mainly discuss cDC1, cDC2 and moDC.

Arguably, cDC1s are an important subset in antitumor immunity, as they are very proficient in antigen uptake and cross-presentation to CD8+ T cells, which leads to the induction of cytotoxic effector T cells and a T_H_1 response (30). cDC2s, although possibly less apt at cross-presentation and priming of CD8+ T cells, are excellent inducers of CD4+ T cells (31). A recent paper by Bosteels et al. showed that inflammatory cDC2s share important characteristics with cDC1s, including potent induction of CD4+ and CD8+ T cell-immunity to viral infections (32). MoDCs are capable in sampling the environment, but are less efficient in migrating to the lymph nodes for activation of CD8+ T cells (31, 33). However, under the right conditions moDCs can activate the antitumor immunity *via* CD8+ T cells (34). Therefore, although moDCs lack clear *de novo* anti-tumor activity, they are also important to consider in anti-tumor therapies.

It is less clear whether any of the circulating DC subsets have a clear-cut tolerogenic function. cDC2s have been described to produce less TNF-α, IL-6, and IL-12 compared to other subsets, together with more IL-10 (35). However, as discussed above, they are also considered potent activators of CD4+ T cell responses. Both mature and immature cDC1s have recently been described as more apt at producing IDO compared to cDC2s (36, 37). However, as stated before, cDC1s are also regarded as important in activating T cells against tumors *via* cross-presentation.

For emphasizing the role of DC subsets in tolerogenic immune modulation it may be more straightforward to look at DC behavior in steady-state tissue. In peripheral tissue, DCs constantly encounter harmless antigens to which an adaptive response has to be dampened (38). Thus, the tissue niche in which DCs reside is an important environmental determinant that shapes the phenotype of DCs further. The skin, for example, harbors epidermal Langerhans cells (LCs), and several dermal DC subsets (DDCs) that seed the skin from cDC2 blood DC progenitors (39). We demonstrated that in contrast to DDCs residing in the deeper dermis layer of skin, epidermal LCs have intrinsically low expression of TLR2, 4 and 5, and accomplish unresponsiveness to innocuous bacteria by limited uptake and presentation of bacterial antigens (38, 40). Similarly, it has been established that under non-inflammatory conditions, CD103+ DC of the gut promote tolerance to harmless commensal bacteria (41, 42). Thus, for the induction of immune tolerance in skin or gut, it may be of therapeutic benefit to target LCs or CD103+ DCs.

It must be emphasized that a clear-cut distinction in pro- or anti-inflammatory specialization of DC subsets in general is difficult. Function of DCs is controlled mainly by stimuli and environmental context, and we can harness this to induce activating or tolerogenic immune responses (17). Therefore, when DCs are targeted with vaccines for either immunity or tolerance, it may be important to filter out beneficial subsets, but it is equally important to provide the right combination of triggers for DCs in order to shape a T cell activating or tolerizing phenotype (43–45).

## Design of a DC-Modulating Liposomal Vaccine Against Cancer, Auto-Immunity or Allergies

**Figure 2:**
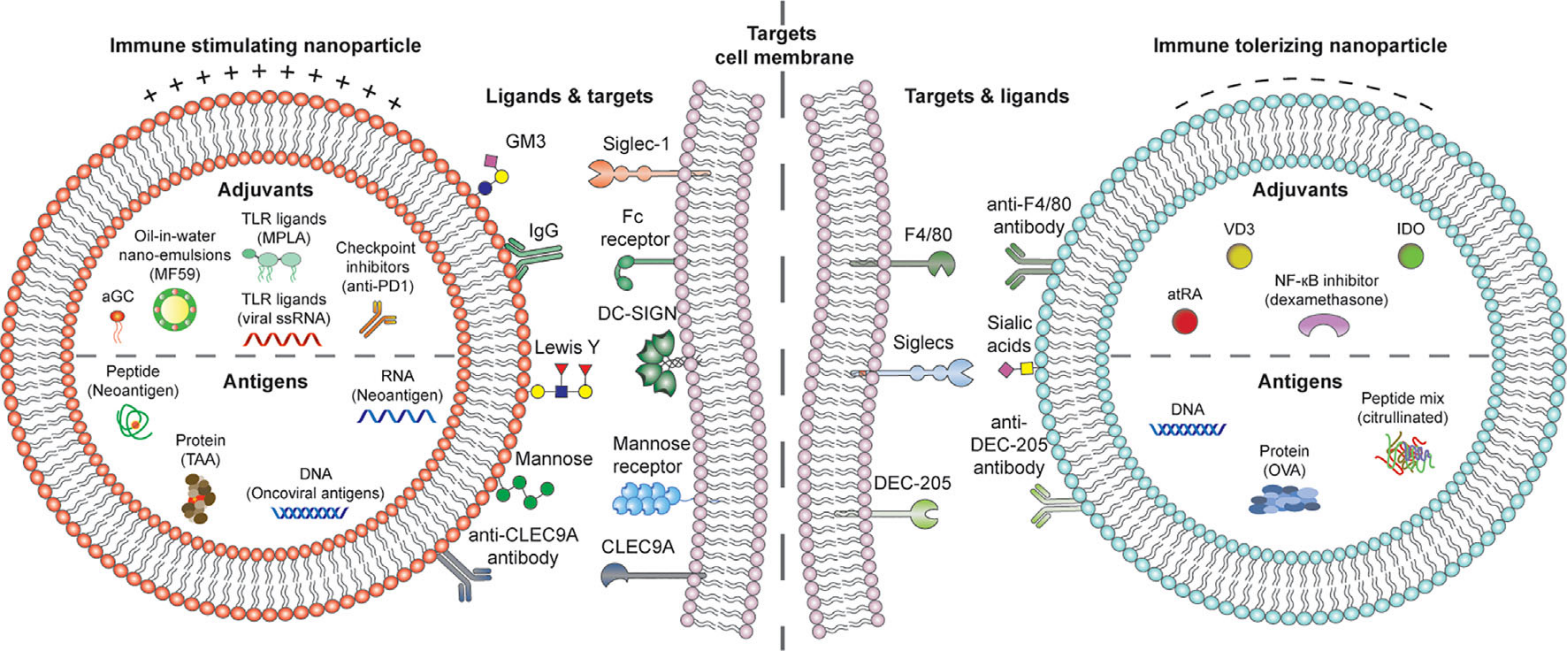
Putative DC-activating cationic (left) or DC tolerizing anionic (right) liposome platforms incorporating disease-specific RNA, DNA, peptide or protein antigens, DC targeting molecules for activation or tolerance, and adjuvants for shaping pro-or anti-inflammatory DCs. αGC, α-galactosylceramide; TLR, toll-like receptor; IgG, Immunoglobulin G; DC-SIGN, Dendritic Cell-Specific Intercellular adhesion molecule-3-Grabbing Non-integrin; atRA, all-trans retinoic acid; VD3, vitamin D3; IDO, indoleamine-2,3-dioxygenase.

For successful DC-based immune modulation with *in vivo* liposome-based vaccines, there are four key components to be considered: a) physicochemical properties of the liposomes, b) disease-specific antigens, c) DC targeting moieties, d) potent adjuvants, with functional properties to induce either immunity or tolerance (**Figure 2**).

Physicochemical properties of nanoparticles are not only important for the stability of the vaccine formulation, but there are also indications in literature that this versatility in design provides DC-modulating and ultimately, general immune modulating opportunities (22).

Disease relevant antigens should be considered in a nano-vaccine platform in order to reprogram antigen-specific adaptive cells against cancer, autoimmunity or allergies, focusing therapies towards modulation of pathogenic immune responses, while leaving the rest of the immune system intact.

Although having the correct antigen is essential in achieving the desired antigen-specific immune response, the quality and magnitude of the immune response also depends on antigen dose. Thus, it is imperative to get sufficient levels of antigens to DCs (46, 47). Targeting nanoparticles to specific surface receptors also induces receptor-specific immune-modulatory effects. Hence, targeting the antigen-carrying nanoparticles to DCs is beneficial for improving specificity of immunotherapy, but also for obtaining durable immune responses *via* receptor-induced immune modulation.

For DC-specific targeting, various surface receptors can be considered. DCs sense pathogen-associated molecular patterns (PAMPs) or danger associated molecular patterns (DAMPS), the ‘flavor’ of a pathogen or the ‘flavor’ of inflammation, *via* PRRs including TLRs, CLRs, NOD-like receptors, Siglecs, and others. The sensing determines the type of polarizing signal expressed by migrant DCs in the lymph node, which may consist of cytokines, membrane-bound or small molecules. In turn, the polarizing signal determines T cell polarization and subset differentiation (48, 49). When triggered, these receptors not only induce or alter various types of (immune) signaling, but in case of CLRs also enhance internalization and (cross-) presentation of the bound molecule (50).

Finally, beyond intrinsic functions of DC subsets and the antigen DCs encounter, the niche, or microenvironment in which this encounter happens seems to be a strong overriding factor for the final outcome of immune modulation (51). Potent adjuvants are therefore needed, that can either revert immune suppression of DCs in the tumor microenvironment or suppress activated DCs in pathogenic inflammation. Loading such potent adjuvants in liposomes may avoid systemic side effects and in conjunction with targeting, focus therapies to the specific disease niche. In the next sections, we will discuss these four key elements of DC-liposome vaccines in context of cancer, allergies and autoimmune disease (**Figure 2**).

## Immune Modulating Properties of Empty Liposomes

### Lipid Composition, Charge, and Rigidity

Various lipids of neutral, positive (cationic) or negative (anionic) electrical charges can be assembled into liposomes with consequences for how they interact with APCs. Positively charged formulations containing lipids, such as 1,2-dioleoyl-3-trimethylammonium-propane (DOTAP), or 3ß-[N-(N’,N’-dimethylaminoethane)-carbamoyl]cholesterol (DC-Chol) have been associated with DC-activating effects. Here, both the net positive electric charge, supporting a favorable interaction with the negatively charged cell membrane, as well as APC interaction with the lipid head groups appear to be important factors for inducing DC activation (52). Several mouse and *in vitro* human studies found an upregulating effect on DC maturation markers and on production of pro-inflammatory cytokines by cationic liposomes (53–55). Due to this assumed adjuvant quality, cationic liposomes are popular candidates for the development of new tumor targeting particulate therapies. Moreover, cationic liposomes appear to be toxic to cells, although a disadvantage when targeting DCs, toxicity is a feature which could be exploited for enhanced tumor cell lysis, thereby also enhancing immunogenicity of cancerous cells (56). Intriguingly, DC-Chol and DOTAP liposomes were found superior in stimulating cross-presentation of OVA to OT-I transgenic CD8+ T cells compared to anionic formulations containing Egg L-α-phosphatidylcholine (EggPC) (57). The authors of that study propose an alkalizing effect of the positively charged formulations on BMDC lysosomes, which leaves OVA more intact for cross-presentation (57). If confirmed by further studies, this would yield another argument for the use of cationic liposomes in anti-tumor targeting. Interestingly, several studies in the context of allergy also employ cationic liposomes, since these formulations proved to be superior in preventing mast cell degranulation and lead to a more efficient reduction in airway eosinophilia and OVA-IgE in allergic mouse models compared to neutrally charged formulations or empty antigen (58, 59).

In the tolerogenic field, liposomes containing the negatively charged lipid, phosphatidylserine (PS) gained considerable attention (22, 60, 61). The most prominent theory for a tolerogenic adjuvant effect of PS containing particles puts forth the notion that such particles resemble apoptotic bodies, thereby silencing DC maturation upon their encounter. Utilizing PS liposomes added to mouse DCs *in vitro*, Shi and colleagues demonstrated that the DCs were resistant to maturation, produced less pro-inflammatory cytokines, acquired the capacity to suppress CD4+ T cell proliferation, as well as to induce PD-1 surface expression on T cells (61). Similar effects were confirmed in a human *in vitro* study, in the context of Type-I diabetes, where patient DCs pulsed with PS-liposomes retained a tolerogenic profile, and suppressed autologous T cell proliferation (62). Interestingly, in a recent study by Benne and colleagues it was not PS containing liposomes, but liposomes incorporating the anionic lipid 1,2-distearoyl-*sn*-glycero-3-phosphoglycerol (DSPG) that induced antigen specific Foxp3+ T cells upon *in vivo* injection in mice (55), the mechanisms of which are yet elusive.

A characteristic that is often altered when changing lipid composition of a formulation is rigidity (22). APCs envelope rigid particles easier than flexible ones leading to more efficient uptake (63–65). In line with these observations Benne *et al.* confirmed enhanced uptake of more rigid variants of the DSPG containing tolerogenic liposomes mentioned above, where injection of more rigid formulations in mice also correlated with stronger Treg responses (66). Similarly, more solid gel-phase pegylated 1,2-distearoyl-sn-glycero-3-phosphocholine (DSPC-PEG) or 1,2-dio- leoyl-sn-glycero-3-phosphocholine (DOPC-PEG) liposomes were taken up better by bone marrow-derived DCs (MDCs) and activated these cells more than their fluid-phase counterparts. Of note, this study used Cholera toxin antigen loaded formulations, and empty formulations did not have an adjuvant effect on BMDCs (67).

### Liposome Size and Shape

Multiple studies emphasize the effect of particle size on uptake mechanisms by APCs, which in turn may influence how liposomal cargo is processed and presented to T cells (22, 68, 69). Similar to how viruses enter cells, particles smaller than 100nm are taken up efficiently by clathrin mediated endocytosis, whereas particles larger than 200nm are phagocytosed or internalized by macropinocytosis (68). This may influence intracellular routing of the liposomal cargo. Cargo escaping the lysosomal route can be more available for cross-presentation on MHC-I, a desirable outcome for DC-mediated cancer therapies but also for tolerizing CD8+ memory T cells employing liposomes with self-antigen (70). Cargo following phagocytosis and the endo-lysosomal route will be preferentially processed towards MHC-II presentation, priming for interaction with CD4+ T cells. Moreover, some studies suggest that particle size has a specific influence on T cell polarization. Nanobeads smaller than 100nm were shown to elicit stronger IFN-γ responses in mice leading to superior T_H_1 immunity compared to beads larger than 100nm (71). As already mentioned small particles may be taken up by DCs similarly to viruses mimicking anti-viral immunity (71). Biodegradable polymer particles in a size range of 1-5µm, on the other hand, were shown to adhere to the DC membrane and offload their cargo there, leading to a T_H_2 response (72, 73). However, conclusions about liposomes seem to point in the opposite direction: liposomes smaller than 200nm were reported to induce T_H_2 immunity whereas larger ones primed towards T_H_1 (74, 75). These observed differences may be explained by uptake and intracellular processing differences between solid particles (such as nanobeads and various polymers) and semi-solid or fluid liposomes. In contrast to solid particles which are taken up by active processes, liposomes can also fuse with the cell membrane and offload cargo directly into the cytoplasm (76). This difference in interaction with DCs can lead to different intracellular processing and presentation of cargo to T cells.

In addition to differences in cellular uptake and processing, size of particles influences bioavailability and biodistribution upon *in vivo* injection. Only particles smaller than 200nm seem to drain to lymph nodes where they can be directly processed by lymph node resident DCs for early T cell activation (22). Larger particles, in contrast, remain at the injection site until phagocytosed by DCs that can migrate to the lymph node, possibly leading to a less vigorous T cell response.

Although less studied, particle shape seems to have an immune modulating effect on APCs as well, where rod-shaped structures (nanorods) were reported as more pro-inflammatory compared to spherical particles (22, 77). However, this feature is less relevant for liposomes as they do not belong to the group of solid nanoparticles.

Despite demonstrated evidence on adjuvant characteristics of certain nanoparticles, a large body of evidence with human cells is lacking. Often several characteristics of a particle are altered at once, making it difficult to discern which characteristic is responsible for observed differences in immunogenicity (22). As visible from the studies discussed in this section, there is also a great need for standardization of different particles or liposomal formulations in order to facilitate a valid comparison between studies. Finally, the therapeutic content loaded in a particle, such as tolerogenic or immune activating adjuvants together with any targeting molecules, may overrule the immune modulatory effect of empty particles (78).

## Battling Cancer With DC Targeted Liposome Vaccines

### Role of Cancer Antigens

Tumors display a wide variety of (abnormally expressed) tumor-associated antigens (TAA), and TAAs such as MART-1, MUC1, WT1, gp100 and the MAGE-A antigens, have been tested in various vaccines in clinical trials (79–82). Using TAA-loaded nanoparticles in different clinical trials have, so far, resulted in mixed responses (83, 84). Since TAA are expressed on both healthy and transformed cells, it is possible that T cells specific for these antigens are deleted during the negative selection in the thymus, which therefore leads to the observed suboptimal anti-tumor responses in many TAA (85, 86). The potential off-target effects induced by targeting TAA make tumor-specific antigens (TSA) an interesting alternative. These antigens are not expressed on healthy cells, and therefore also have limited tolerance related complications.

TSA include mutated neoantigens, but also antigens from endogenous origin. In particular, because ~12% of human cancers are caused by viruses, the foreign (viral) antigens expressed on the transformed cells are highly immunogenic (87, 88). The VGX-3100 vaccine, targeting the HPV proteins E6 and E7, the main oncoviral antigens of the cancer caused by the virus, is based on a DNA vaccine of the mentioned antigen, and is currently tested in a phase III clinical trial (NCT03185013) (89). Parallel to this non-liposomal vaccine liposomal (archaesomes) delivery systems for DNA encoding the HPV antigens are being tested, and show the induction of a potent anti-tumor immune response in an *in vivo* cancer model (90). Another class of TSA arise from non-synonymous DNA mutations, and are therefore called neoantigens (91). These antigens are highly immunogenic and therefore highly sought after targets for therapeutic vaccines (92). Since the neoantigens are personal to the patient, they will need to be identified per patient through genomic comparison of tumor and normal tissue (93). Recent personalized clinical trials with vaccines targeting neoantigens feature high immune activation and overall promising results (94, 95). Nanoparticles are already used to deliver the neoantigens (e.g. RNA, DNA or peptides) to DCs *in vitro, in vivo* and in clinical trials (85, 96, 97). Especially for (m)RNA vaccines, nanoparticles such as liposomes are greatly beneficial since they protect the payload against degradation (98). Accordingly, a recent phase I clinical trial with patient personalized tumor mRNA-loaded nanoparticles showed high tolerability, and the interim results from another recent phase I clinical trial showed encouraging clinical responses (NCT03897881) (99). Exploiting the same pharmacological advantage, the recently successful mRNA based SARS-CoV-2 vaccines have a nanoparticle delivery system (100, 101).

### Targeting DCs *In Vivo* for Immune Activation

PRRs researched for targeting of DCs in context of cancer therapy include CLRs, the Siglec receptor Siglec-1, and Fc receptors (FcR). CLRs recognize carbohydrate ligands, which makes them important sensors of differently glycosylated PAMPS (102). Some of these receptors are expressed on a broad class of APCs, whereas others are DC or even DC-subset specific. Thus, targeting of these receptors by adding glycan moieties or CLR antibodies to liposomes can be used for reaching APCs with a varying spectrum of cell specificity. DC-SIGN is a CLR expressed on different subsets of APCs, including moDCs, CD14+ dermal DCs, subsets of macrophages and DCs at mucosal sites (103). Targeting liposomes to DC-SIGN *via* its natural glycan ligand Lewis Y showed increased CD8+ T cell responses *in vitro *and* ex-vivo (* 104). Also, targeting the DC-SIGN receptor with antibodies conjugated to nanostructures, leads to increased immune responses (105). Unfortunately, a clinical trial with a DC-SIGN targeting nanoparticle vaccine Lipovaxin-MM has not resulted in immunogenic anti-tumor responses (106). The vaccine’s antigens (e.g. gp100 and MART-1) were derived from plasma membrane vesicles from a human melanoma cell line, and modified with a liposomal mixture, also containing IFN-γ. A DC-SIGN targeting antibody was also incorporated in the membrane, allowing for targeting to various APCs. While the vaccine was well tolerated, significant immunogenicity of the vaccine was not detected. Similarly to DC-SIGN, the mannose receptor (MR) is also expressed on various APCs, such as macrophages and moDCs. Targeting of MR has already been evaluated in a clinical trial, proving that the administration of the MR-targeting vaccine together with local application of TLR agonists, induces significant humoral and cellular immune responses (107). When mannosylated nanoparticles were used to target the MR, it led to high effector T cell responses and reduced tumor growth *in vivo (* 108). Immunization of mice with liposomes made of mannose-mimicking ligands loaded with DNA encoding for MART-1 allowed for efficient transfection of CD11c+ DCs, inducing long lasting melanoma specific prophylactic CTL responses (109). Therapeutic vaccination with these liposomes resulted in delayed tumor growth in mice. In contrast to the previously discussed CLRS, DEC-205 appears more DC-specific with expression demonstrated on cDC1s, cDC2s and moDCs. When DCs were targeted with PLGA nanoparticles coupled to monoclonal anti-DEC205 antibodies, the treatment lead to enhanced internalization, cross-presentation and CD8+ T cell activation (110). Similar results were observed by another group, showing that targeting of nanoparticles to DEC-205, CD40 or CD11c improved priming of cytotoxic CD8+ T cells over untargeted nanoparticles (111). Targeting CLEC9A, a CLR with advantageously restricted expression on cDC1 DCs, also elicited anti-tumor responses in multiple studies, coherent with the potent (cross-)presenting function of these DCs (112, 113). In an additional study, targeting PLGA nanoparticles loaded with the TAA Trp2 and gp100 to CLEC9A expressing DCs *via* antibodies resulted in strong therapeutic anti-tumor responses *in vivo*, but also induced *in vitro* expansion of NKT and CD8+ T cells specific for melanoma in PBMCs from both healthy donors and melanoma patients (114).

Other groups of receptors that have been used for targeting APC in the context of cancer are Siglecs and FcR. While most Siglecs confer tolerogenic responses, the Siglec-1 (or CD169) receptor, expressed on splenic macrophages is of special interest for anti-tumor responses. These cells transferred antigen to cross-presenting cDC1s when targeted with liposomes coated with the Siglec-1 ligand GM3, conferring beneficial anti-tumor CD8+ T cell responses (115). FcR, which bind to the constant domains of antibodies can be targeted by coating liposomes with antibodies. Indeed, IgG coated liposomes bearing the OVA antigen prevented development of OVA-expressing lymphoma, in contrast to the liposomes without IgG coating (116). Also, specifically the Fc fragment of an antibody can be used on the outside of a nanoparticle for targeting purposes, which induced increased cellular and humoral immune responses in mice when a cancer peptide was included in the nanoparticle (117).

Targeting one receptor may in several cases induce either immunity or tolerance, depending on the vaccine formulation and microenvironment. For instance, targeting DC-SIGN can display T_H_2 polarizing effects, combined with inhibition of T_H_1/T_H_17, when targeted with natural ligands (118). Accordingly, depending on the adjuvants used, DEC-205 targeting is used for both tolerogenic and immunogenic purposes (119). Therefore, when targeting DCs *via* specific receptors, it is not only important to target the appropriate receptor, but also to provide efficient co-stimulatory adjuvants to properly skew the immune response (51).

### Vaccine Adjuvants for Immune Activation

TLRs are a well-known class of PRRs, and some of these are targeted for induction of antitumor responses (120). Molecules targeting TLR4 are LPS structures derived from bacteria, but as adjuvant the less toxic variant of LPS, monophosphoryl lipid A (MPLA) is used. The incorporation of MPLA in PLGA nanoparticles increases T_H_1 and pro-inflammatory responses in comparison to the non-encapsulated administration of MPLA (121). Agonists for TLR7/8 are viral ssRNA and synthetic compounds like R848. Triggering TLR7 for cancer therapy with Imiquimod has already been approved by the FDA, and clinical trials with the TLR7/8 stimulating adjuvant R848 have been conducted (120, 122). The prophylactic and therapeutic effect of OVA mRNA-loaded nanoparticles as vaccine against OVA expressing lymphoma in an *in vivo* mouse model is increased when R848 is added, and results in increased TAA presentation and antigen specific CD8+ T cell populations (123). The TLR9 stimulating ligand CpG has also been evaluated in clinical trials, with positive outcomes (124). When CpG is incorporated into a nanoparticle, its efficacy in terms of (delayed) tumor growth was superior in comparison to the soluble form of CpG, again highlighting the potential of nanoparticles for antigen and adjuvant delivery (125). TLR3, recognizing dsRNA, is another PRR receptor that is targeted for pro-inflammatory responses (126). The synthetic analogue of TLR3, Poly (I:C), is being evaluated as adjuvant in (pre-) clinical trials, and elicits favorable effects (94). Thereby, encapsulation of the TLR3 adjuvant in a nanoparticle further potentiates the inflammatory immune responses (127). Especially when Poly (I:C) is combined with the TLR9 stimulant CpG in a nanoparticle, it induces protective and therapeutic immune responses in *in vivo* models (128, 129). Other combinations of TLR ligands in nanoparticles are also used, with established synergistic effects (120, 130). Hence, it is becoming clear that incorporating multiple TLR stimuli in liposomes will be a promising adjuvant strategy for immunogenic vaccines.

An adjuvant that works directly *via* presentation on DCs is α-galactosylceramide (αGC), a potent immune activating glycolipid which when presented on DCs, initially activates iNKTs (131). In turn, these iNKTs activate other NK-, CD8+-T, B-cells and DCs, *via* increased cytokine production (132). Since αGC is a glycolipid, it also allows for easy incorporation in- for example- liposomal nanoparticles. Incorporated in liposomes, αGC leads to the increase in CD8+ T cell responses *in vitro (* 104). Accordingly, αGC incorporated in liposomes is able to reduce the outgrowth of lung melanoma metastasis *in vivo (* 133).

Another way to increase vaccine efficiency is by increasing the numbers, and immunological state, of immune cells in the site of vaccination. The adjuvant (MF59) used in the seasonal influenza vaccine, based on an oil-in-water nano-emulsion, increases the number of APCs and creates an immunogenic microenvironment (134). Enhancement of APC numbers also improves trafficking of antigens to the draining lymph nodes *via* leukocytes, favoring a stronger immunogenic response. Oil-in-water nano-emulsions are able to encapsulate both antigens and antibodies, for specific targeting to for instance the CLEC9A receptor, thereby becoming self-adjuvating delivery systems for DC vaccination (135).

## Fostering Immune Tolerance With DC-Targeted Liposome Vaccines

### Role of Disease Specific Antigens in Immune Tolerance

Repeated exposure to antigen has shown tolerance inducing effects. AIT exploits this very concept with curative success demonstrated for various pollen and venom allergies (3). However, treatment efficacy for other allergies, such as food allergies, could be increased, together with reduction of risk of anaphylaxis by incorporating allergens in nanoparticles. A clinical study with subcutaneous injection of liposomes incorporating house-dust mite extract was already carried out as early as 2002 (136). Unfortunately, this study did not compare treatment with soluble allergen and no clear line of clinical studies followed. Even though the validity of allergic mouse models is somewhat questionable, several recent studies demonstrate therapeutic proof-of concept. In a mouse model of pollen allergy to the weedy plant *Chenopodium album*, subcutaneous injection of allergen incorporating protamine-DNA liposomes shifted a predominant T_H_2 response to the allergen in a T_H_1 direction, with decrease of IgE, increase in IgG2a and IFN-γ production specific to allergen (137). Chaisri et al. tested effects of intranasal vaccination with liposomes incorporating Derp1 and Derp2 separately, or in combination (138). Interestingly, even though all formulations reduced T_H_2 immune reactions, only the liposomes incorporating single allergens lead to expression of tolerogenic cytokine genes TGF-β, IL-35 and IL-10 in mouse lung cells. In an OVA mouse model of allergy, sublingual treatment with OVA incorporating liposomes preceding allergen challenge was superior to treatment with free OVA (59). Unfortunately, none of the above studies examined airway hyperresponsiveness as a read-out, which could make the conclusions about treatment efficacy stronger.

In contrast to allergies where the antigen is known and clearly defined, autoimmune conditions pose the difficult challenge of unknown causative auto-antigens or foreign antigens that may trigger disease. In rheumatoid arthritis (RA), anti-citrulline antibodies (ACPA) appear in blood before disease onset and can be very specifically linked to RA pathogenesis (139). This knowledge stimulated a quest for putative, citrullinated antigens on cartilaginous surfaces capable of stimulating auto-reactive T cells. Indeed, using a panel of HLA-DRB1*04:01 tetramers, James and colleagues confirmed an increased presence of citrullinated antigen specific T cells in peripheral blood of RA patients compared to healthy subjects (140). Of note, Benham and colleagues performed immunotherapy using *ex vivo* RA patient-derived moDCs with an NF-κB inhibitor and a mix of citrullinated antigens (141). This (uncontrolled) treatment strategy proved safe and showed some signs of efficacy such as a decrease in effector T cells and improvement in clinical RA scores. As early as 2009, the same research group established that citrullinated antigens loaded in EggPC liposomes can efficiently be used to induce antigen-specific Foxp3+ Tregs in mice, an effect that was specifically mediated by DCs (142).

### Targeting DCs *In Vivo* for Tolerogenic Immune Modulation

Several CLRs and Siglecs are under scrutiny for targeting, as an attempt to specify tolerogenic *in vivo* DC therapies. For an extensive review on this subject we refer the reader to a very recent piece published by our colleagues (21). The CLR DEC-205 is highly expressed on the cross-presenting cDC1 DC subset (as well as on cDC2s and moDCs) and is therefore a widely studied receptor for nanoparticle DC targeting, for example *via* antibodies (143). Targeting the DEC-205 receptor *via* antibodies without providing maturation stimuli can lead to specific induction of T-cell anergy as well as increased T cells suppression (119). OVA-loaded PLGA nanoparticles that were conjugated with antibodies to DEC-205 induced IL-10 production in DCs and subsequently T cells, of which the levels were dependent on the amount of antibodies on the nanoparticles (144). However, the T_H_1 priming of DCs targeted with these nanoparticles was not impeded.

The F4/80 receptor, expressed on macrophages and DCs, shows promise in inducing tolerance in a nonobese diabetic mouse model. The progression of diabetes in the *in vivo* model was prevented by vaccinating mice with liposomes coated with anti-F4/80 antibodies and a disease specific short peptide joined with a TLR-2 ligand (145).

Modification of antigens with the carbohydrate ligands (sialic acid) of Siglecs expressed by DCs results in the induction of antigen specific Tregs and alleviation of allergic symptoms in mice (146, 147). Other studies focus on nanoparticles targeting Siglecs to induce tolerance in B-cells or other immune cells, but to date, no studies have been reported using nanoparticles that target to Siglecs on DCs (148, 149).

Similarly to allergies, there is pre-clinical precedent for the use of nanoparticles for developing new treatment modalities against RA and other autoimmune diseases. However, studies with a targeting component towards DCs are not abundant in literature, highlighting a knowledge gap that needs to be addressed.

### Vaccine Adjuvants for Immune Tolerance

In order to prevent adverse reactions to allergens or a worsening of autoimmunity it is highly likely that co-delivery of tolerance inducing compounds together with disease relevant antigen will be a necessary element of successful *in vivo* targeting therapies. Indeed, multiple studies observed immunogenic reactions to nanoparticles delivering antigen only (69, 150–152). This may also apply to AIT utilizing nanocarriers. For example, in a mouse study of cockroach allergy, only allergen encapsulated in liposomes together with a tolerogenic adjuvant induced increased transcription of IL-10, TGF-β and IL-35 as well as IDO1 (153).

The choice of adjuvants with the strongest immune dampening effect may be critical for successful DC-mediated tolerance *in vivo*. The vitamins D and A have been extensively studied in the tolerogenic context. 1,25alfa-dehydroxycalciferol, or vitamin D3 (VD3) appears to be the most potent immunosuppressant of all forms of vitamin D (154, 155). Both mouse and human studies have demonstrated that VD3 priming of immature and mature DCs results in a tolerogenic phenotype with induction of co-inhibitory receptors, reduced IL-12 production and induced IL-10 secretion (156–160). The tolerogenic effects of VD3 have also been shown in several skin-derived DC subsets where priming of *in vitro* cultured LCs, CD1a+ DDCs or skin-derived DCs with VD3 resulted in the outgrowth of distinct Treg phenotypes (161, 162). Most importantly, VD3-raised DCs show tolerogenic stability in face of repeated rechallenge with pro-inflammatory stimuli, making VD3 a robust DC-tolerizing candidate in an already inflamed environment (163). In line with that, several recent studies in mice demonstrated that nanoparticles loaded with VD3 and OVA induced tolerogenic DCs with *in vitro* and *in vivo* suppressive capacity of OVA-specific T cells (164, 165). Additionally, subcutaneous injection of VD3-loaded particles resulted in effective targeting of PD-L1 high draining lymph node DCs, resulting in amelioration of a RA disease model (165).

In contrast to the well-established tolerogenic role of VD3, there is still considerable debate on whether the active form of Vitamin A, all-trans retinoic acid (atRA) has pro- or anti-inflammatory effects (166). Similarly to murine mucosal CD103+ DCs (167), human moDCs raised with atRA, induce the development of IL-10 producing Tr1 in co-culture (41). Building on the anti-inflammatory potential of atRA, the compound was recently incorporated in PLGA particles together with atherosclerosis autoantigen and improved atherosclerotic lesions *in vivo (* 168). In a further recent study, atRA was encapsulated together with another anti-inflammatory adjuvant, triptolide, in galactose-containing nanoparticles (78). *In vivo* effects in mice consisted of reduced infiltration of these sites by T cells and pro-inflammatory macrophages, together with reduced expression of T_H_1-T_H_17 polarizing cytokines in inflamed tissue extracts. However, atRA also supports induction of T_H_1 and T_H_2 responses upon inflammatory stimulation, serving as one explanation of contradictory pro- and anti-inflammatory effects observed (169, 170). Thus, loading of atRA in nanoparticle platforms for tolerogenic purposes may be an interesting option, but with a sidenote of caution.

A central transcription factor downstream of activating signals delivered to DCs is NF-κB, which makes it one of the key targets for immune modulation. Corticosteroids and glucocorticoids are well-known to exert their immunosuppressive effects *via* NF-κB inhibition (171, 172). Treatment of moDCs with the corticosteroid dexamethasone leads to an immature phenotype with loss of IL-12 secretion and high IL-10 secretion. Similarly to VD3 treated DCs, dexamethasone DCs seem robustly maturation resistant and capable of inducing IL-10 producing Tregs, although these Tregs exert suppression in a non-antigen specific manner (173). To prevent systemic immune suppression, loading of corticosteroids into nanoparticles and targeting the particles only to cells relevant for inflammation is a treatment approach considered in RA and other autoimmune conditions, showing promising treatment efficacy in several mouse studies (174). In addition to these rather a specific inhibitors of NF-κB signaling, also several highly specific inhibitors of (non)canonical signaling have been investigated in human moDCs and demonstrated to potently reduce T cell responses (175). Recently, also VD3 was confirmed to downmodulate NF-κB signaling in human moDCs matured with LPS, providing an elegant bridge between VD3 and NF-κB inhibition as adjuvants (176).

Indoleamine 2,3-dioxygenase (IDO), the enzyme responsible for breaking down the essential amino acid tryptophan into kynurenine can be produced by DCs, which leads to decreased T cell proliferation, induction of Tregs and an anergic T cell phenotype in co-culture experiments (177–179). IDO production in the gut is intricately intertwined with the gut microbiota, and exerts tolerogenic effects on CD103+ DCs as well as dampening tissue T_H_1/T_H_17 responses (180–182). Several molecules may induce IDO production by DCs, however, some of these, such as type I interferons or IFN-γ are also pro-inflammatory cytokines, hindering their use in tolerance inducing therapies (183). Induction of IDO has been described upon CD40 ligation of moDCs, where the enzyme was induced by non-canonical NF-κB signaling (36). After treating mouse pDCs with TGF-β, IDO was activated as a downstream signaling molecule, leading to inhibitory signaling and the activation of the non-canonical NF-κB pathway, further strengthening IDO expression in a self-feeding loop (184). Further compounds that were demonstrated to induce IDO are soluble CTLA-4, the TLR-agonists LPS and CpG, together with DNA agonists of stimulator of interferon genes (STING) (183). As treatment with TLR-agonists carries pro-inflammatory risks, soluble CTLA-4 and STING agonists seem better suited candidates for DC-treatment. Indeed, nanoparticles constituted of CpG free pDNA were demonstrated to induce IDO *via* the STING pathway, leading to amelioration in experimental autoimmune encephalomyelitis (183). Moreover, this therapeutic effect could be strengthened when blocking downstream metabolites of IDO additionally to DNA nanoparticle treatment.

In addition to the above compounds discussed in detail, several other materials are tested for their tolerogenic potential, such as various parasite-derived antigens, plant-derived adjuvants or compounds already well-known in the clinic, like the m-TOR inhibitor rapamycin (185). In fact, just like VD3 and dexamethasone, rapamycin was demonstrated to induce robustly tolerogenic, clinical grade DCs (186). Although rapamycin loading into several forms of nanoparticles is actively tested for better delivery to cancer cells or as an inflammation dampening adjuvant, targeting to DCs has not been in the focus of research thus far (187).

## Outlook

Based on the discussion presented in this review an ideal immune modulating nanoparticle DC-vaccine should harbor the following properties: a) physicochemical characteristics promoting tolerance or activation, b) antigen relevant for the given disease condition to create disease specificity c) specific targeting molecules aimed at tolerogenic or pro-inflammatory DCs, and d) tolerogenic or immune activating adjuvants (**Figure 2**). For unravelling exact immune modulatory properties of all vaccine components, further fundamental studies will be needed, featuring a careful comparison of nanoparticle characteristics, with a stepwise selective approach towards the most optimal particle-targeting-adjuvant and antigen combination for DC-therapy. It is clear that the field of nanomedicine is in need for standardized research, carried out with similar methodology, using similar nanoparticles.

Apart from immune modulating elements, the advantage of nanoparticles lies in the ability to synergistically combine multiple characteristics and compounds to achieve a desired DC-tolerizing or activating outcome. Indeed, ongoing clinical trials conducted with DC-targeting nanoparticles, combining targeting, antigen and adjuvants in the context of cancer and several mouse studies in autoimmune or allergic disease leave cause for optimism. However, beyond advantages, several key aspects need more consideration during development of novel nanomedical treatments. Cationic liposomes have cytotoxic effects as they disrupt cell membranes, a possible advantage when targeting cancer cells directly but not when targeting APCs (76). While solid polymer particles are generally stable, semi-solid liposomes can be unstable depending on their surface chemistry. Neutral lipids or cationic formulations, for example, are known to aggregate quickly due to lack of electrostatic repulsion or rapid attraction of negatively charged proteins to their surface (188). In fundamental studies, liposomal formulations are often used within two weeks of manufacturing and opinions on their stability differ (55). Clearly, studies need to monitor stability more strictly and for a longer period of time. Furthermore, hydrophilic cargo such as peptide or protein antigens, as well as other chemical components, such as fluorescent lipid dyes loaded onto the surface of liposomes tend to dissociate (189). Dependent on the nature of the cargo this could lead to undesired bystander effects, deleterious side effects such as anaphylaxis in case of allergen loading, and misinterpretation of molecular results, such as of cellular uptake and intracellular processing (189). The potential disadvantages carefully need to be assessed in the context of each specific disease setting.

As highlighted earlier, for the induction of a potent anti-tumor response, it is crucial to use DC targeted vaccines that will provide enough antigen and co-stimulation for the DC to mount an inflammatory response (190). Since nano-formulations can provide all four components, a plethora of research is focused on these platforms. So far, pre-clinical data has shown promising results in favor of using DC-targeting nanoparticle formulations for the induction of potent anti-tumor responses. While nanotechnology for directly targeting DCs for anti-cancer immunotherapy is mainly applied in *in vivo* and *ex-vivo* models, some clinical trials are conducted with this platform (106, 191). Unfortunately, liposomes targeting the DC-SIGN receptor in patients with metastatic melanoma did not yield desired clinical results (106). The authors note that it is not the lack of anti-melanoma immunity that may cause absence of treatment efficacy, but rather the suppression of (pre-existing) anti-melanoma immunity. This notion can be extrapolated to other immunogenic tumors as well, suggesting the use of additional components in the vaccine platform in order to boost pre-existing anti-tumor immunity (192). The promising research into checkpoint inhibitors (CI) could provide another combinational treatment option for cancer, since CI effectiveness is in large part dependent on the pre-existing anti-cancer immunity (193, 194). Of note, the combination of checkpoint inhibitors, and nanoparticles incorporating TLR agonists and peptides, showed strong synergistic effects in *in vivo* mouse models for the treatment of cancer (195, 196). Therefore, combining CI with a (nanoparticle based) therapy aimed to initiate/reinvigorate the anti-tumor response, should be strongly considered (197, 198).

Similarly to the cancer field, the stage is set for clinical trials of *in vivo* targeting of DCs to treat inflammatory diseases. In a much awaited clinical study in RA research, the previously mentioned EPC liposomes incorporating the NF-κB inhibitor BAY11-7082 and citrullinated peptides are injected in RA patients for antigen-specific inhibition of pro-arthritic immune responses (142). For a successful therapeutic outcome in tolerogenic applications essential factors will be co-delivery of disease relevant antigens with an immune-dampening adjuvant in order to avoid adverse pro-inflammatory effects (69). In the same line, adjuvant cargo will have to be carefully chosen based on pre-existing studies demonstrating ability to induce robustly tolerogenic DCs that withstand the immunogenic temptations of highly inflammatory environments (163). Route of administration should also be studied further as it can potentially play a role in tolerogenic effects. For example, only when applied as an intranasal vaccine and not intra-muscularly, did OVA-loaded PLGA particles promote transcription of FoxP3 in cervical lymph nodes (199).

To date, no *in vivo* DC-targeting nanoparticle vaccine is available in the clinic, but the promising mRNA-based nanoparticle vaccines for SARS-CoV-2 and new results from ongoing nanoparticle-based cancer clinical trials, as well as preclinical studies in autoimmune diseases are expected to accelerate research into the platform (200).

Based on existing evidence presented in this review, it is certain that collaborations, synchronization of nanomedical experimental practices as well as the accumulation of data in human cells and clinical studies will bring a new wave of promising research strengthening the potential of DC-based treatments for cancer, allergies and autoimmunity.

## Author Contributions

NN and AH performed the literature search, wrote the manuscript, and created all figures. EJ and YK critically read and carefully revised all versions of the manuscript providing valuable guidance and insight. TG, RR, and ST critically read the manuscript and provided valuable additions. All authors contributed to the article and approved the submitted version.

## Funding

This work was supported by LSH-TKI project DC4Balance LSHM18056-SGF.

## Conflict of Interest

The authors declare that the research was conducted in the absence of any commercial or financial relationships that could be construed as a potential conflict of interest.
